# Effects of Outer Membrane Vesicle Formation, Surface-Layer Production and Nanopod Development on the Metabolism of Phenanthrene by *Delftia acidovorans* Cs1-4

**DOI:** 10.1371/journal.pone.0092143

**Published:** 2014-03-18

**Authors:** Ameesha Shetty, William J. Hickey

**Affiliations:** O.N. Allen Laboratory for Soil Microbiology, Department of Soil Science, University of Wisconsin-Madison, Madison, Wisconsin, United States of America; University of Florida, United States of America

## Abstract

Nanopods are extracellular structures arising from the convergence of two widely distributed bacterial characteristics: production of outer membrane vesicles (OMV) and formation of surface layers (S-layers). Nanopod production is driven by OMV formation, and in *Delftia acidovorans* Cs1-4 growth on phenanthrene induces OMV/nanopod formation. While OMV production has been associated with many functions, particularly with pathogens, linkage to biodegradation has been limited to a membrane stress response to lipophilic compounds. The objectives of this study were to determine: 1.) Whether induction of nanopod formation was linked to phenanthrene metabolism or a non-specific membrane stress response, and 2.) The relative importance of OMV/nanopod formation *vs.* formation of the S-layer alone to phenanthrene utilization. Membrane stress response was investigated by quantifying nanopod formation following exposure to compounds that exceeded phenanthrene in membrane stress-inducing potential. Naphthalene did not induce nanopod formation, and toluene was a weak inducer compared to phenanthrene (two- *vs*. six-fold increase, respectively). Induction of nanopod formation by growth on phenanthrene was therefore linked to phenanthrene metabolism and not a membrane stress response. Impacts on phenanthrene biodegradation of OMV/nanopod production *vs.* S-layer formation were assessed with *D. acidovorans* Cs1-4 mutants deficient in S-layer formation or OMV/nanopod production. Both mutants had impaired growth on phenanthrene, but the loss of OMV/nanopod production was more significant than loss of the S-layer. The S-layer of *D. acidovorans* Cs1-4 did not affect phenanthrene uptake, and its primary role in phenanthrene biodegradation process appeared to be enabling nanopod development. Nanopods appeared to benefit phenanthrene biodegradation by enhancing cellular retention of metabolites. Collectively, these studies established that nanopod/OMV formation was an essential characteristic of the *D. acidovorans* Cs1-4 phenanthrene degradation process. This report thus established a new dimension in the area of biodegradation, namely, the involvement of extracellular structures as elements supporting metabolic processes underlying biodegradation.

## Introduction

Nanopods are recently-discovered extracellular structures [1] that arise from the convergence of two widely-distributed bacterial characteristics: production of outer membrane vesicles (OMV) and formation of surface layers (S-layers). Nanopods consist of strings of OMV encased within a tubular sheath of a novel glycosylated, surface layer protein (SLP) termed Nanopod Protein A (NpdA). Nanopods develop from points on the outer membrane that are loci of repetitive OMV release, and ≥180 OMV can be generated from a single such locus yielding nanopods that are ≥6 μm long [1]. Thus, OMV production is an important mechanism driving nanopod formation, and these structures are effectively conduits for OMV deployment. Nanopods were first observed in phenanthrene-degrader *Delftia acidovorans* Cs1-4 (formerly *Delftia* sp. Cs1-4), but production of NpdA and nanopod production appears to be a characteristic of *Delftia*, *Acidovorax* and other genera in the family *Comamondaceae*. Furthermore, some marine *Gammaproteobacteria* possess NpdA orthologs and produce nanopods [1], and nanopod-like structures have also been observed in archaea [2]. Thus, nanopods appear to be fundamental structural component of diverse prokaryotes that form S-layers.

Prior work with *D. acidovorans* Cs1-4 indicated growth on phenanthrene induced OMV/nanopod formation [1], and while OMV production has been associated with a wide variety of bacterial functions [3], [4], [5], [6], [7], linkage to biodegradation has been limited to a membrane stress response [8], [9]. Membrane stress can result from partitioning of hydrophobic compounds into lipid bilayers, and the potential of such compounds to induce membrane stress increases with increasing aqueous solubility (decreasing octanol-water partition coefficient, K_OW_) with compounds with logK_OW_ ≤ 4 having the greatest potential for toxicity [10]. Phenanthrene is outside of the high toxicity range (logK_OW_ ≈ 4.5), but although it would appear to be a relatively weak inducer of membrane stress, the potential for phenanthrene to illicit some level of stress that might drive OMV formation cannot be excluded. Alternatively, instead of a relatively non-specific stress response, induction of OMV production by growth on phenanthrene could reflect a more specific association with that process. For example, OMV can package small molecules that mediate cell signaling and nutrient acquisition [3], [4], [5], [6], [7] and could be linked to phenanthrene metabolism.

While the S-layer has an essential role as a structural component of nanopods [1], other potential linkages to phenanthene degradation, such as enhancing substrate uptake, are unknown. For *D. acidovorans* Cs1-4, an impact of the S-layer on uptake is possible, as chemical properties of the cell surface can strongly affect uptake [11], [12], and the S-layer rather than outer membrane lipopolysaccaride (LPS) would determine surface characteristics of the cells (and nanopods). The effects of varying LPS content on cell surface hydrophobicity and uptake of hydrophobic compounds, primarily antibiotics, have been extensively investigated [12], [13], [14], [15], [16]. In contrast, relatively little is known about impacts of S-layers on uptake of hydrophobic substrates. Kay et al. [17] reported binding of water soluble compounds (Congo red, protoporphyrin and hemin) to an SLP from *Aeromonas salmonicida*, and found binding was more effective to cells lacking the S-layer. However, effects of this S-layer on interactions with hydrophobic compounds were not examined. Also, the *A. salmonicida* SLP lacked glycosylation [18] that, if present, could affect surface characteristics in the same manner as LPS.

In prior work [1], structural characteristics of nanopods were elucidated and initial evidence was gained that linked OMV/nanopod formation to growth on phenanthrene. The present study built on those findings, and focused on exploring the potential linkages of OMV/nanopod production and S-layer formation to phenanthrene degradation by *D. acidovorans* Cs1-4. Mutants of *D. acidovorans* Cs1-4 were utilized that lacked the S-layer, yet retained OMV production [1], or retained the S-layer, but were diminished in OMV/nanopod production [19]. The objectives were: 1.) To establish the extent to which OMV/nanopod production was a membrane stress response *vs*. a process linked to metabolism of phenanthrene, and 2.) To determine the relative importance of S-layer formation and OMV/nanopod production to the utilization of phenanthrene by *D. acidovorans* Cs1-4.

## Materials and Methods

### Bacterial strains and culture conditions

Bacterial cultures used were *D. acidovorans* Cs1-4 wild type (WT), mutant M3 and mutant M6 (Table S1). Also, a transposon mutant of *D. acidovorans* Cs1-4 that was flagella-free, but retained wild type levels (Table S1), was used in the experiments examining membrane stress and those on OMV/nanopod production. Cultures were routinely grown in mineral salts medium (MSM, [20]) with phenanthrene (1 mg/mL; Sigma-Aldrich, St. Louis, MO) as the sole source of carbon and incubated at 28°C with shaking at 150 rpm. For selected experiments, sodium pyruvate (Sigma) was added to MSM as the carbon source instead of phenanthrene. All cultures were started with an inoculum containing 10^8 ^cells/mL pre-cultured on phenanthrene, and were established in triplicate for quantitative analyses.

### Analysis of OMV and nanopod production

Phenanthene-grown cultures were passed through a glass fiber filter (Millipore, Billerica, MA) to remove phenanthrene crystals, and the filtrate passed twice through a 0.2 μm Durapore membrane (Millipore). The resulting cell-free filtrate was centrifuged (20,000 x *g*, 1 h) and the pellet of the extracellular fraction was resuspended in 500 μL sterile phosphate buffer (PB; 10 mM, pH 7.0). Aliquots of this preparation were taken for protein analyses and imaging by transmission electron microscopy (TEM), and the remainder used in immunoassays. Cellular and extracellular protein profiles were examined by sodium dodecyl sulfate polyacyrlamide gel electrophoresis (SDS-PAGE) as described previously [1]. Protein yields were measured by using the Pierce bicinchoninic acid kit (Thermo Scientific, Rockford, IL), and the data were analyzed for significant differences (*p*≥0.05) by applying the Students t-test. For imaging by TEM, samples were centrifuged at 20,000 x *g* for 1 h, and then visualized by negative staining with uranyl acetate with a JEOL 100CX 100 kV transmission electron microscope.

Antibody (IgY) against whole nanopods (SLP and OMV) was produced as described previously [1]. The extracellular fraction preparation was labeled with anti-nanopod IgY (1∶1000) at 37 °C for 10 min. This was followed by centrifugation at 20,000 x *g* for 1 h. After two washings with sterile PB, the pellet was labeled with the secondary antibody (1∶500) DyLight 488 anti-IgY conjugate (Jackson Immunoresearch, West Grove, PA) for 10 min at 37°C. This step was followed by centrifugation at 20,000 x *g* for 1 h, followed by two more washes with sterile PB. The final pellet was resuspended with 200 μL PB, and fluorescence was quantified using a Synergy microplate reader (Biotek, Winooski, VT). The resultant values were normalized to the protein content of the sample, and then used as an index of nanopod production. The data were analyzed for significant differences (*p*≥0.05) by applying the Students t-test.

### Membrane stress experiments

The flagella-free *D. acidovorans* Cs1-4 culture was grown on MSM-pyruvate with and without toluene (600 μg/mL) or naphthalene (1 mg/mL). Triplicate cultures (200 mL) were grown in Fernbach flasks (sealed with parafilm) with shaking at 120 rpm. When cultures reached stationary phase, they were harvested for extracellular protein and immunoassay analyses as described above. The data were analyzed for significant differences (*p*≥0.05) by applying the Students t-test.

### Phenanthrene uptake

[9-^14^C]Phenanthrene (2.04 GBq mmol^−1^; purity, 99.6%; Sigma) and [^12^C]phenanthrene (Sigma) were mixed in hexane (Sigma) to give a ^14^C/^12^C stock of 25 μg total phenanthreneμL^−1^ (specific activity, 7.4 Bqμg^−1^). Cultures (500 mL) of *D. acidovorans* Cs1-4 WT and mutant M3 were grown with phenanthrene as the carbon source. The cells were harvested at late log phase by centrifugation at 7,000 x *g* for 15 min. The pellet was then washed and re-suspended in phosphate buffered saline (PBS) to OD_600_ ≈ 0.1. Uptake tests were done as in prior work [21]. To start the assay, the ^14^C/^12^C stock was added to 4 mL of cell suspension to give a final phenanthrene concentration of 5.6 μM (below the aqueous solubility limit of phenanthrene of *ca.* 7 μM). Aliquots (1 mL) were removed from the reaction mixture at 15 s intervals over a period of 1 min. Cells were filtered onto 0.45 μm nitrocellulose membrane (Millipore), and washed twice with PBS. Heat-killed cells were used as a negative control [21]. The filters were then collected, and added to scintillation vials with 10 mL Scintisafe scintillation cocktail (Fisher Scientific, Pittsburgh, PA). Radioactivity on the filters was measured using a Tri-Carb 2100TR liquid scintillation analyzer (Packard Instrument company, Meriden, CT). The uptake rates were compared for significant differences (*p*≥0.05) by analysis of covariance (ANCOVA) using the R programming environment (R-project.org).

## Results and Discussion

### Nanopod/OMV production and membrane stress

For both the *D. acidovorans* Cs1-4 WT and *D. acidovorans* Cs1-4 flagella-free mutant there was a significant correlation between the protein content and immunoreactivity of the extracellular fraction (*p* < 0.001). The protein content and immunoreactivity of extracellular fractions from *D. acidovorans* Cs1-4 WT and the *D. acidovorans* Cs1-4 flagella-free mutant were not significantly different. Thus, flagella were a relatively minor constituent of the extracellular protein, and the extracellular fraction was dominated by nanopod-associated protein (*i.e*., S-Layer and/or OMV). Protein content and immunoreactivity were thus good indicators of nanopod production for both *D. acidovorans* Cs1-4 WT and the flagella-free mutant. As phenotypes of these two cultures were similar with respect to nanopod production, the latter was used in the membrane stress experiments. This was done to eliminate the possibility that exposure to substrates other than phenanthrene might induce flagella production, and potentially make flagellar protein a significant, but unknown, fraction of the total extracellular protein.

There was no correlation between levels of extracellular protein content or immunoreactivity and logK_ow_ of the hydrocarbon to which the culture was exposed. Compared to cultures grown on pyruvate alone, the extracellular fraction of pyruvate-grown cultures exposed to naphthalene (logK_ow_ ≅ 3.3) showed no significant difference in protein content or immunoreactivity. Cultures exposed to toluene (logK_ow_ ≅ 2.7) exhibited a 2-fold increase in protein content and immunoreactivity relative to cultures grown on pyruvate alone. But, the effect of toluene was significantly (*p*<0.001) less than the 6-fold increase in extracellular protein content and immunoreactivity effected by phenanthrene (logK_ow_ ≅ 4.5, [1]).

It was hypothesized that, if OMV/nanopod production was primarily a response to membrane stress, then compounds having stress-inducing potential that was equal to, or greater than, that of phenanthrene should induce a commensurate level of OMV/nanopod formation. Theoretically, the membrane stress-inducing potential of toluene and naphthalene exceeded that of phenanthrene, as their higher aqueous solubility would have resulted in greater cellular exposure to these hydrocarbons and greater uptake into membranes. Furthermore, toluene was not metabolized by *D. acidovorans* Cs1-4, so confounding effects that might result from formation of potentially stress-inducing metabolites (*e.g*., phenolics) were eliminated.

Induction of OMV/nanopod production by growth on phenanthrene was thus concluded as not reflecting a membrane stress response such as that occurring in other bacteria when exposed to hydrocarbons [8], [9]. Instead, induction of OMV/nanopod production was likely linked to metabolism of phenanthrene. The studies also indicated that metabolism-linked induction of OMV/nanopods was specific to phenanthrene; as a different growth-supporting substrate, pyruvate, added at an available carbon level similar to phenanthrene, did not induce formation of these structures. Specificity to phenenthrene was also indicated as naphthalene, a different polynuclear aromatic hydrocarbon that was cometabolized by *D. acidovorans* Cs1-4, did not induce OMV/nanopod formation. Thus, findings of the present studies established a connection of OMV production to hydrocarbon biodegradation that extended beyond the stress response that has been established in the literature.

### Production of OMV and nanopods by mutant cultures

The phenotypes of mutant M3 and mutant M6 for production of OMV/nanopods were divergent, and established by three independent lines of evidence. First, the extracellular fractions were analyzed quantitatively for variation in protein content and immunoreactivity. Yields of extracellular protein from mutants M3 and M6, were reduced by 66% (483 ± 52 μg protein/L) and 96% (59 ± 8 μg protein/L) respectively, relative to WT levels (1421 ± 95 μg protein/L). The amounts of extracellular protein produced by both mutants were significantly less than WT levels (*p* ≤ 0.05), and amounts of protein produced by mutant M3 were significantly greater than those of mutant M6 (*p* ≤ 0.05). Both mutants showed significant reductions in immunoreactivity relative to WT levels, with mutant M3 reduced by 70% and mutant M6 reduced by 94% (*p* ≤ 0.05). For each mutant, the reductions in the immunoreactive fraction was significantly correlated with the relative reductions in total extracellular protein (*p* ≤ 0.05).

A second line of analysis for characterization of mutants for production of OMV/nanopods was TEM imaging of the extracellular fractions. Nanopod production characteristic of phenanthrene-grown *D. acidovorans* Cs1-4 was exhibited by the flagella-free mutant, which showed an abundance of nanopods, but no free OMV (Figs. S1 A-C in File S1). In contrast, the extracellular fraction of mutant M3 was devoid of nanopods, but contained many free OMV (Figs. S1 D-E in File S1). Mutant M6 contained nanopods, but these were truncated, and at levels much reduced relative to the WT (Figs. S1 G-I in File S1). Mutant M6 also contained some OMV, although their abundance was less than that in cultures of mutant M3 (Figs. S1 G-I in File S1).

**Figure 1:**
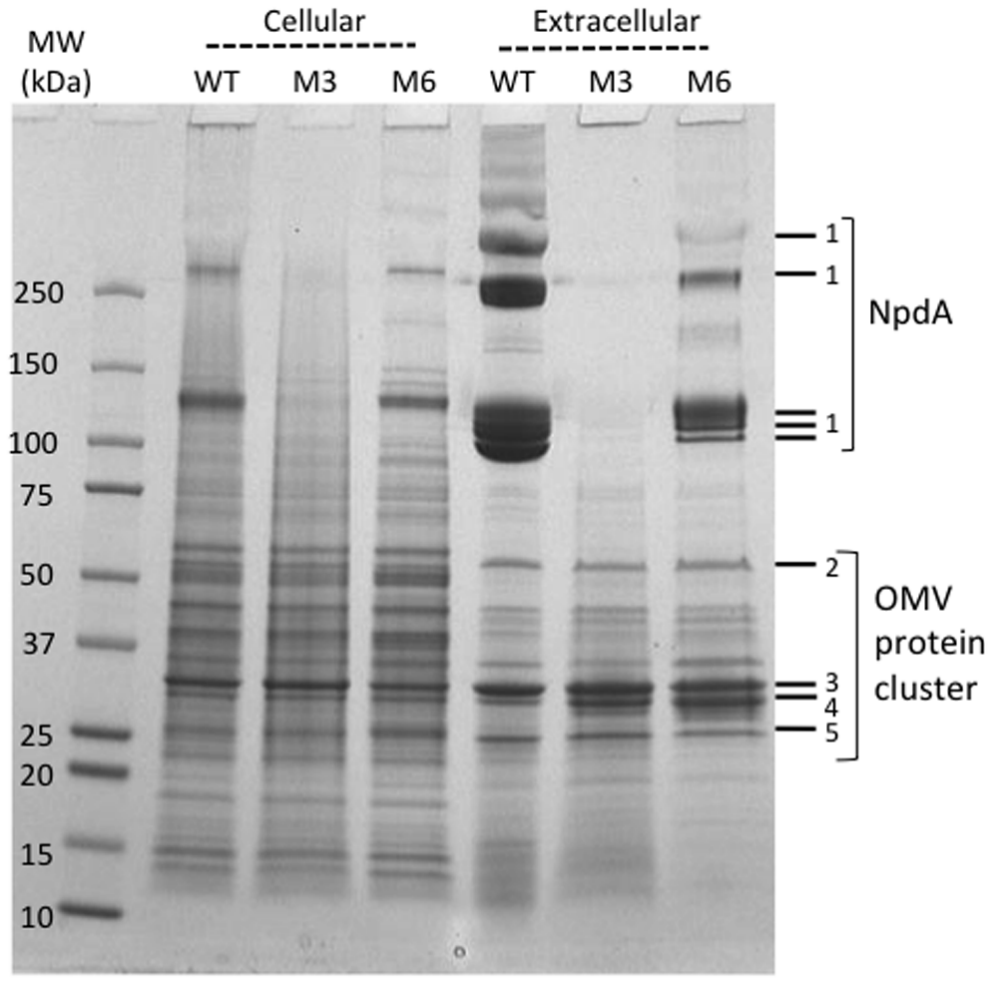
Protein profiles of cellular and extracellular fractions of *D. acidovorans* Cs1-4 wild type (WT), mutant M3 (M3) and mutant M6 (M6). Brackets indicate oligomers of the surface layer protein (NpdA) or clusters of proteins associated with outer membrane vesicles (OMV). Numbers indicate proteins identified by Shetty et al. [1] as: 1.) NpdA, 2.) Protein with domain of unknown function 1302, 3.) OmpC-like protein, 4.) Omp32, 5.) OmpA-like protein. Separation by SDS-PAGE was done in a 4–20% Tris-HCl gel with a total of 30 μg protein loaded in each sample lane.

Lastly, mutants were characterized for production of OMV/nanopods by SDS-PAGE profiles of extracellular fractions. The protein profile of *D. acidovorans* Cs1-4 WT showed the typical abundance of the SLP (NpdA), with its characteristic laddering pattern and the cluster of OMV-associated bands (Fig. 1). The extracellular profile of mutant M6 was similar to that of the WT, except that the relative intensity of the SLP bands was reduced (Fig. 1). For this mutant, the reduced intensity of the SLP bands *v.* OMV cluster was consistent with TEM images showing free OMV and nanopods. Thus, the presence of OMV in the extracellular fraction would increase the intensity of the OMV bands relative to those of the SLP. In contrast, the profile of extracellular protein for mutant M3 displayed the OMV cluster, but not the SLP bands (Fig. 1). As the SLP was the dominant constituent of the WT extracellular fraction, the reduced yield of extracellular protein from mutant M3 probably reflected to a large degree the absence of the SLP.

These data established that while mutant M3 lacked the SLP and was unable to form nanopods, it retained the capacity for phenenthrene-induced OMV formation. In contrast, disruption of *phnL* in the phenanthrene gene cluster of mutant M6 [19], [22] greatly diminished OMV production, and consequently, nanopod development. The predicted product of *phnL* (locus, Delcs14_1758) was identified only as containing the Pfam domain of photosynthesis system II assembly factor YCF48, which in cyanobacteria is needed for assembly and repair of photosystem II membrane proteins [23], [24]. While the function of the PhnL is unknown, its relation to a protein involved in membrane structure could be relevant to processes affecting OMV formation. Moreover, *phnL* orthologs occur in a diversity of proteobacteria, and have a common feature of association with hydrocarbon oxidoreductase systems, particularly oxygenases [22]. Thus, it’s possible that a broad function of *phnL* is the linkage of OMV production to some hydrocarbon metabolism pathways.

Regardless of the genetic mechanisms underlying OMV/nanopod formation, the phenotypes of mutants M3 and M6 enabled evaluation of the relative importance of S-layer formation *vs.* OMV/nanopod production in phenanthrene degradation. In this regard, as mutant M3 retained OMV/nanopod production, impacts on phenanthrene metabolism by this strain could be attributed to absence of the S-layer. Conversely, as mutant M6 retained the S-layer, but was disabled in OMV formation, alterations in phenenthrene degradation would indicate a role for OMV/nanoods in that process.

### Phenanthrene degradation and uptake by wild-type and mutant cultures

**Figure 2:**
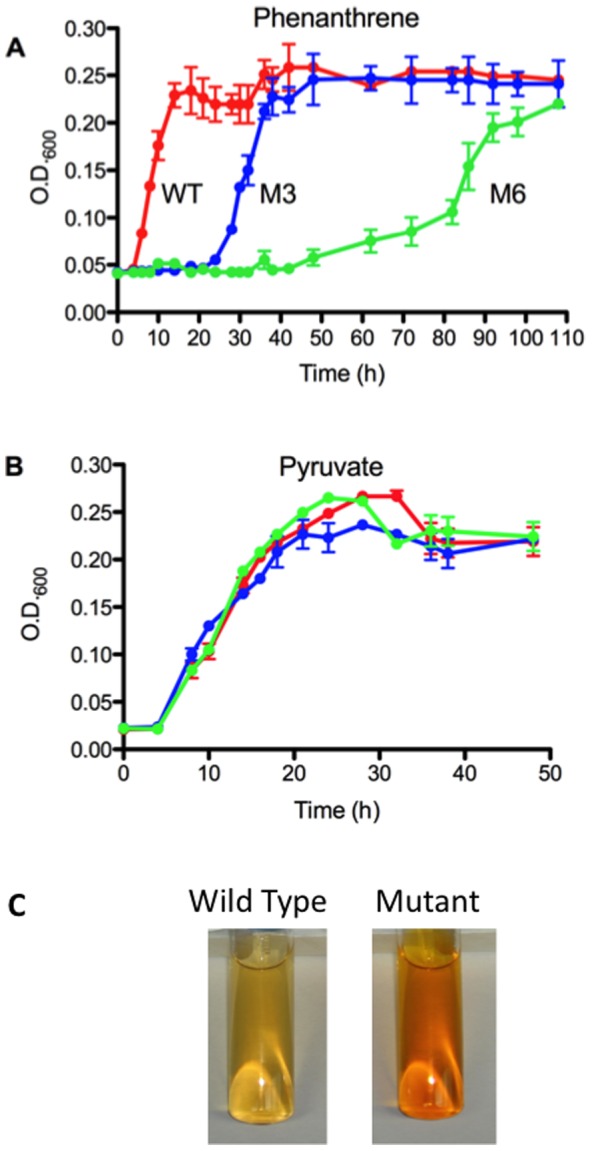
Growth curves of *D. acidovorans* Cs1-4 wild type, mutant M3 and mutant M6 on phenanthrene (Panel A) and pyruvate (Panel B). Each data point is the mean of measures for triplicates of each culture and bars represent standard error of the mean. Panel B. Example of medium coloration for *D. acidovorans* Cs1-4 wild type (WT) and mutants M3 and M6.

Mutant M3 and mutant M6 both showed impaired growth on phenanthrene (Fig. 2A). For mutant M3, impairment was reflected in an extended lag phase of 21 h *vs.* a 6 h lag time for the WT (Fig. 2A). Mutant M6 showed an even greater lag time of 48 h, and also showed a diminished growth rate (Fig. 2A). In contrast, growth on pyruvate by mutant M3 and mutant M6 was indistinguishable from that of the WT (Fig. 2B), indicating that the impaired growth of mutants reflected alteration of a cellular characteristic that was important to growth on phenanthrene, but not pyruvate, and thus probably did not affect an aspect of basic metabolism. Also, since all cultures were pre-grown on phenanthrene, the extended lag phase of the mutants was probably not explained by enzyme induction or other aspects of acclimation.

**Figure 3:**
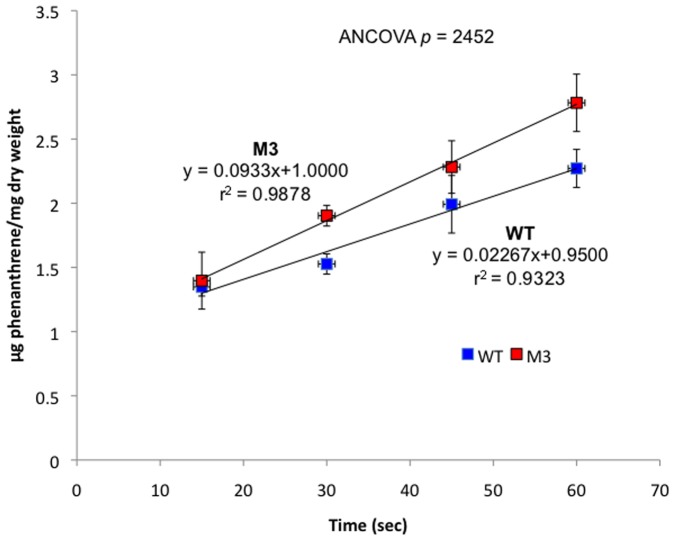
Uptake of phenanthrene by *D. acidovorans* Cs1-4 wild type (WT) and mutant M3 (M3) cells. Each data point is a mean of triplicates and error bar represent standard error of the mean. Data are corrected for background measured in control assays.

For mutant M3, absence of the S-layer would expose the underlying LPS, and consequently change properties of the cell surface that might affect phenanthrene uptake. However, uptake of [^14^C]phenanthrene by mutant M3 and the WT was not significantly different (ANCOVA *p*  =  0.2452, Fig. 3). Thus, there was no evidence that the S-layer had a significant role in phenanthrene uptake. Consequently, it’s unlikely that either diminished phenanthrene uptake or heightened phenanthrene uptake (*i.e.*, toxicity) was a factor contributing to the extended lag phase of mutant M3.

Growth of both mutants on phenanthrene differed from the WT in exhibiting extracellular accumulation of metabolites, which was indicated by a yellow-orange coloration of the medium (Fig. 2C). These accumulations occurred quickly, and were typically visible within *ca.* 2 h following inoculation with either mutant M3 or mutant M6. Metabolite secretion probably did not reflect a generalized increase in cell leakage resulting from loss of the S-layer, as mutant M6 retained that structure. However, metabolite secretion did more closely parallel disruption of OMV association with cells and/or with other OMV. In the WT, packaging of OMV into nanopods maintained close proximity between OMV, as well as between OMV and the cell. In contrast, free OMV released by mutants M3 and M6 maintained no steady association with either the cells or other OMV. Possibly, such contact is needed to prevent leakage of compounds secreted within OMV. If so, a key function of nanopods may be deployment of OMV in a structure that facilitates molecule retention, and the primary importance of the S-layer in phenanthrene biodegradation by *D. acidovorans* Cs1-4 would be enabling nanopod formation. This hypothesis could also at least partly explain the enigmatic role of S-layers in general, as the S-layer may not be functionally significant in and of itself (*e.g.*, facilitating attachment, uptake *etc.*). Instead, its importance is derived from an interaction with another cellular structure (*i.e.*, OMV) and giving rise to a new entity (*i.e.*, nanopod) that is beneficial to the cell.

The reason(s) why molecule retention might be important in the phenanthrene degradation process are unclear, but it could be that cells are able to recover secreted molecules from OMV and derive a benefit from those compounds. While OMV have been most extensively studied for their protein cargo, secretion of small molecules within OMV has also been established. For example, in *Pseudomonas aeruginosa*, the quorum-sensing/iron-binding compound *Pseudomonas* quinolone signal and the bacterocin pyocin are secreted in OMV [5], [25], [26], [27]. Thus, secretion of metabolites within OMV would be a possibility for *D. acidovorans* Cs1-4.

Fluids from cultures of the WT and both mutants contained phenanthrene, 1-hydroxy-2-naphthaldehyde, *o*-phthalate and an unknown metabolite. Culture fluids of mutant M3 and mutant M6 contained three unidentified compounds absent in the profile of the WT, and two of these compounds were determined by mass spectrometry and boron-11 nuclear magnetic resonance to contain boron. None of the unidentified metabolites were detected in pyruvate-grown cultures of the WT, mutant M3 or mutant M6. The identity and function of the boron-containing metabolites produced by *D. acidovorans* Cs1-4 is as yet unknown. But, detection in phenanthrene-grown cultures, and not those grown on pyruvate, indicated an association with phenanthrene degradation. Further detailed structural analysis will be needed to identify this compound, and potentially gain insights into its function.

## Conclusions

For *D. acidovorans* Cs1-4, nanopod/OMV formation was an essential characteristic of the phenanthrene degradation process, as production of these structures was induced by metabolism of phenanthrene, and mutants disabled in nanopod/OMV formation were unable to grow effectively on this compound. The S-layer of *D. acidovorans* Cs1-4 did not affect phenanthrene uptake, and its primary role in phenanthrene biodegradation process appeared to be enabling nanopod development, structures that appeared to benefit phenanthrene biodegradation by enhancing cellular retention of metabolites linked to phenanthrene catabolism. The present report thus established a new dimension in the area of biodegradation, namely, the involvement of extracellular structures as elements supporting metabolic processes underlying biodegradation.

## Supporting Information

File S1 **Figure S1.** Negatively stained cultures imaged by TEM showing extracellular material in phenanthrene-grown cultures of *D. acidovorans* Cs1-4 WT (Panels A-C), mutant M3 (Panels D-F ) and mutant M6 (Panels G-I). Panel A: Nanopods in the WT culture varying in length from *ca.* 50 nm (box) to ≥1500 nm (arrow). Panel B: Magnified view of WT nanopods showing linear structures (arrow) and segments (box). Panel C: Magnified view of WT nanopods showing cross-hatched surface structure indicative of paracrystalline S-layer. Panel D: Extracellular environment of mutant M3 containing OMV (boxes) and flagella (arrows) but devoid of nanopods. Panels E and F: Magnified view of OMV and flagella in the mutant M3 culture, with OMV ranging in size from *ca.* 20 nm to 100 nm. Panels G and H: Extracellular environment of mutant M6 containing nanopods (boxes), OMV (circles) and flagella (arrows). Nanopods were sparse compared to the WT, and truncated with none more than *ca*. 500 nm in length. Panel I: Magnified view of mutant M6 culture with truncated nanopods (boxes), OMV (circles) and flagella (arrows).
(PDF)

Table S1 ***Delftia acidovorans*** **Cs1-4 strains used in this study.**
(DOCX)
