# Supplementary figures and images for: Effects of Outer Membrane Vesicle Formation, Surface-Layer Production and Nanopod Development on the Metabolism of Phenanthrene by Delftia acidovorans Cs1-4
Source: PLoS One. 2014 Mar 18;9(3):e92143. doi: 10.1371/journal.pone.0092143 (PMC3958437; doi:10.1371/journal.pone.0092143)

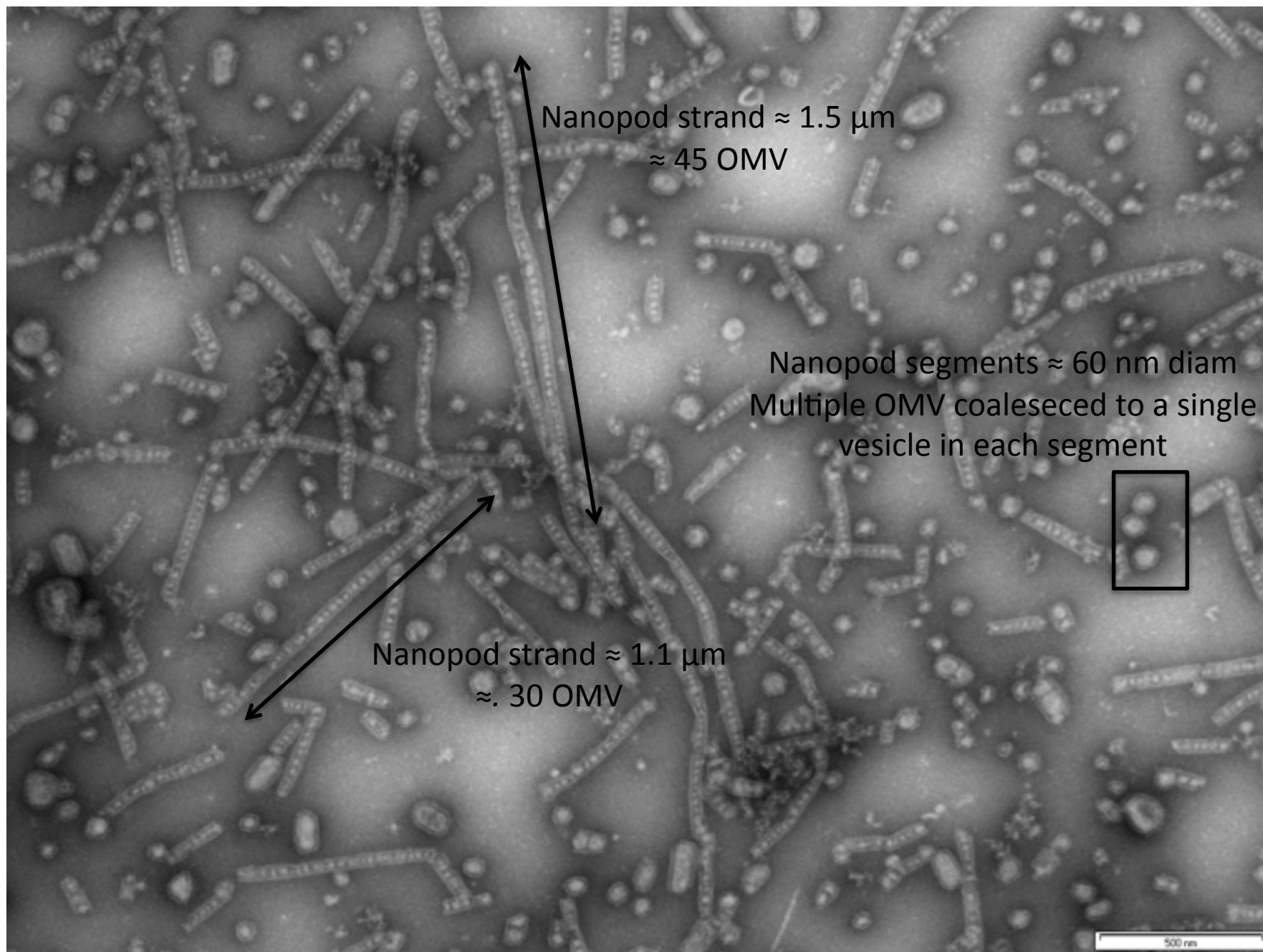

Figure S1\_A

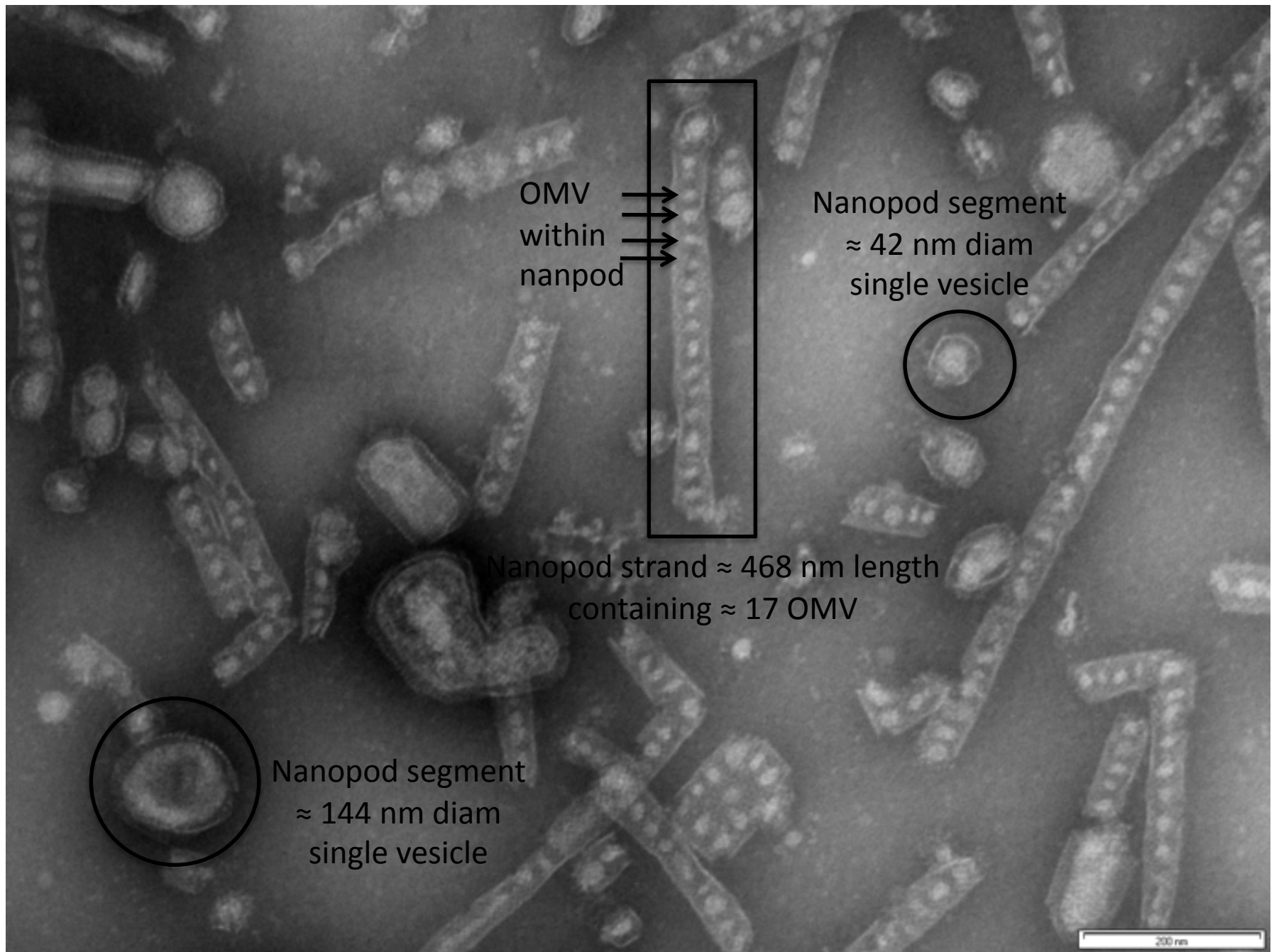

Figure S1\_B

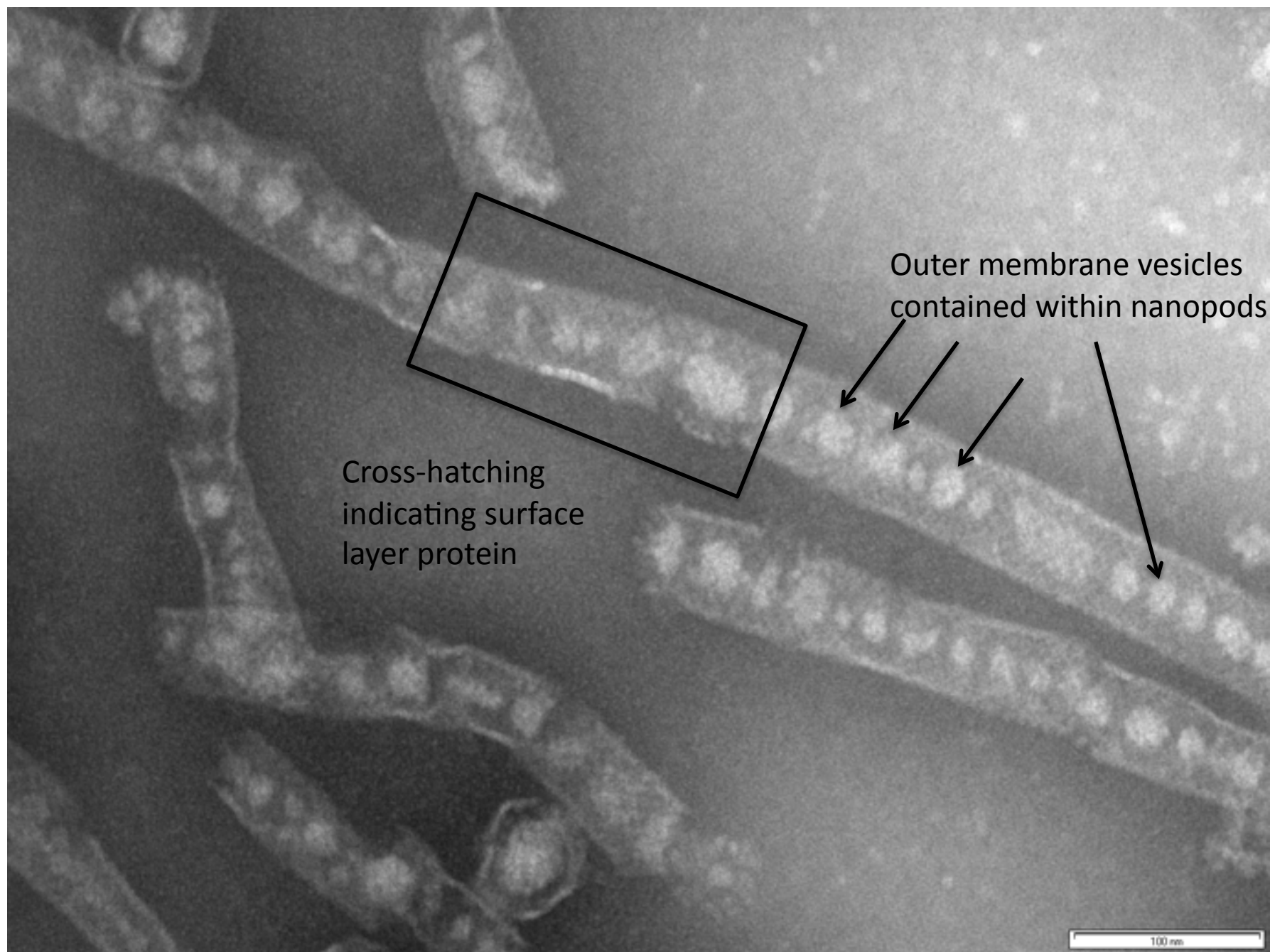

Figure S1\_C

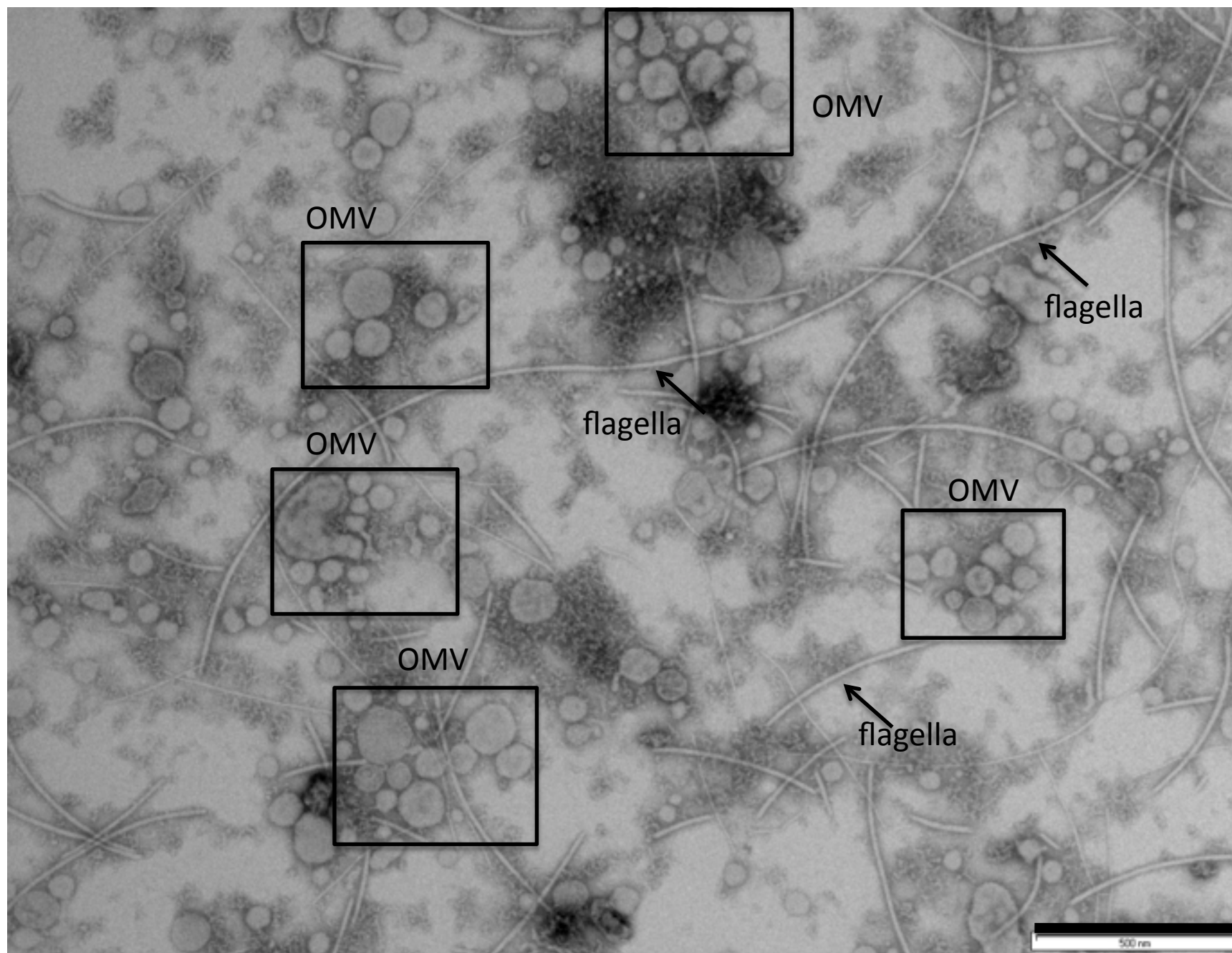

Figure S1\_D

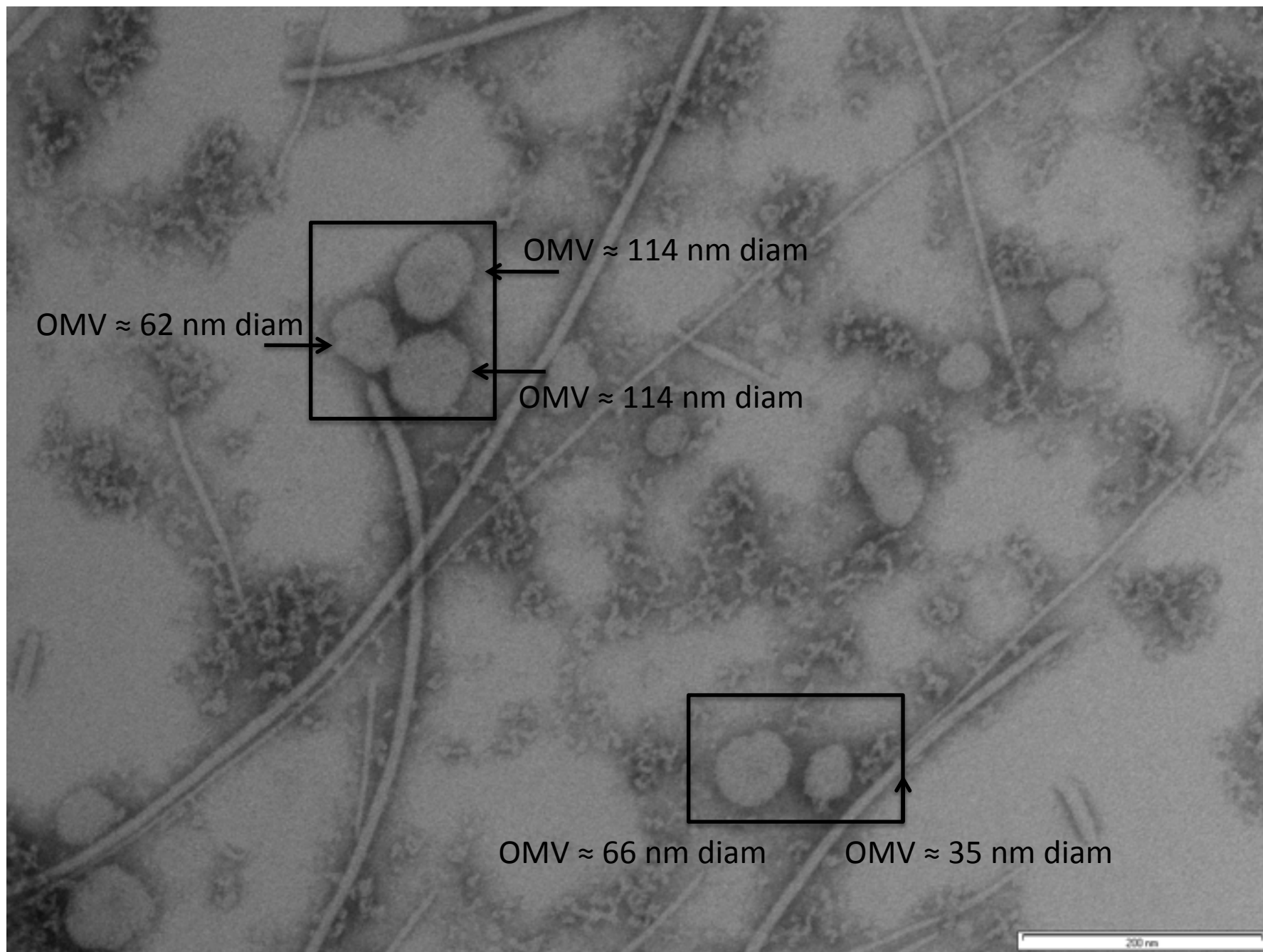

Figure S1\_E

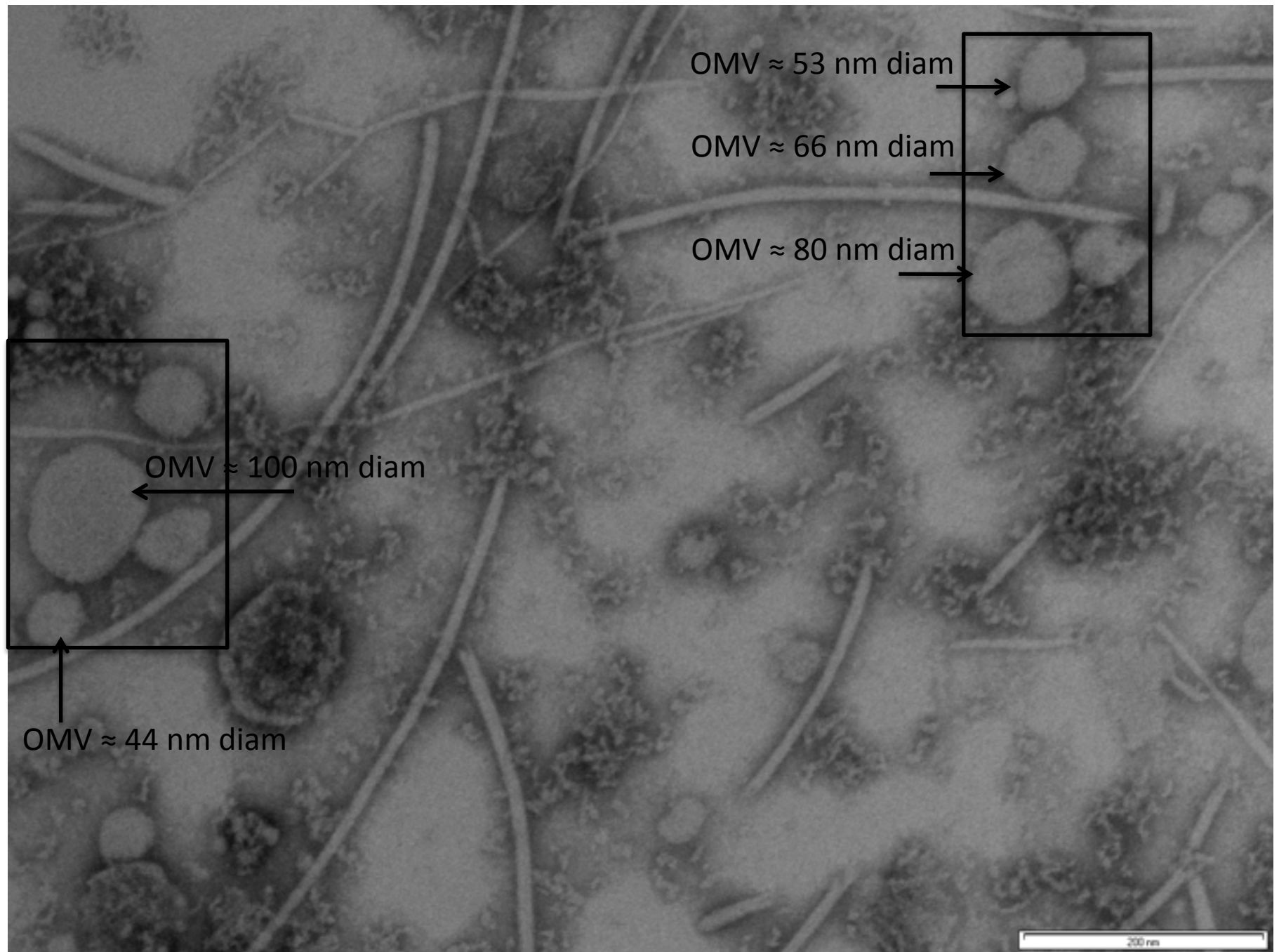

Figure S1\_F

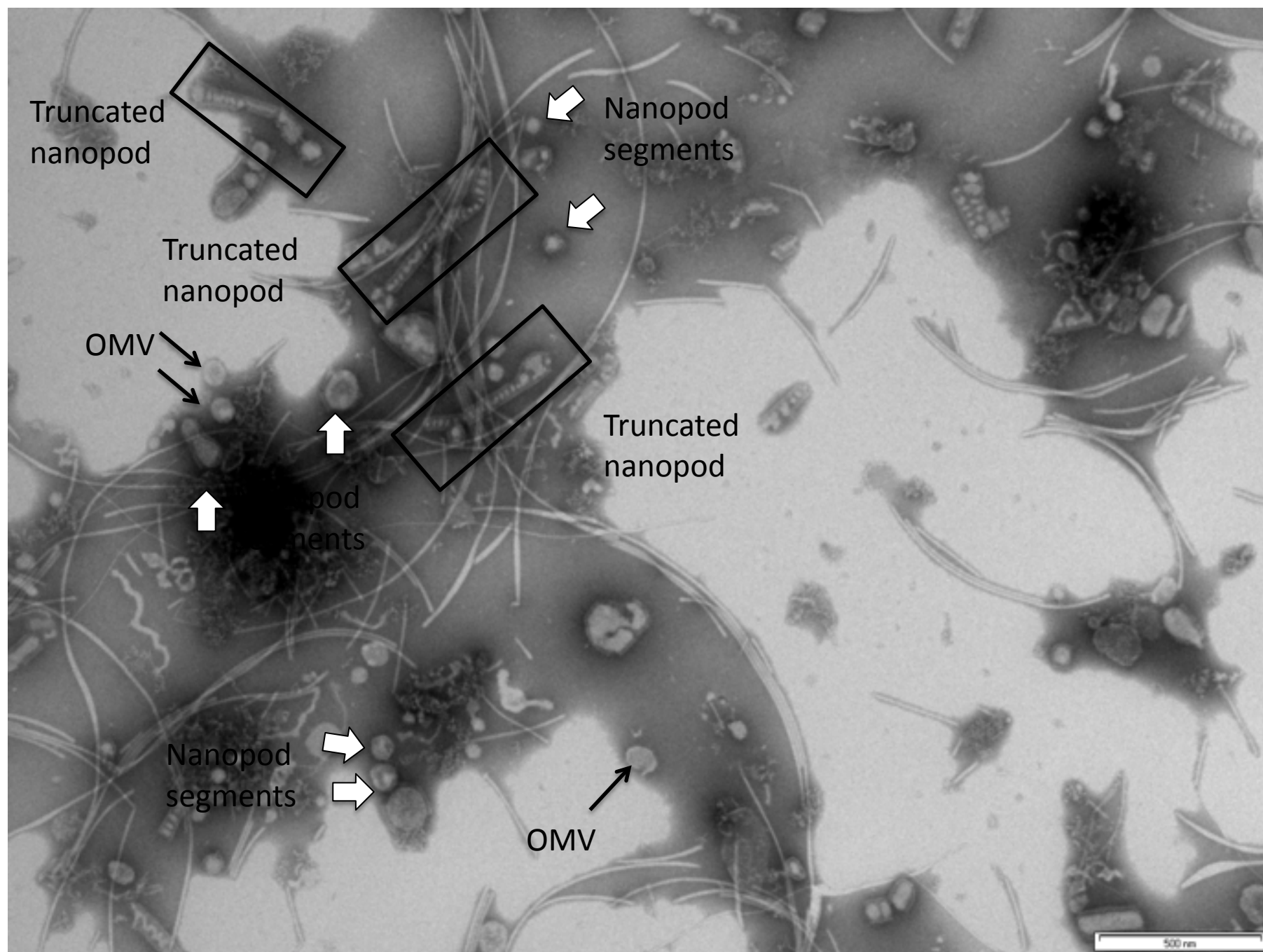

Figure S1\_G

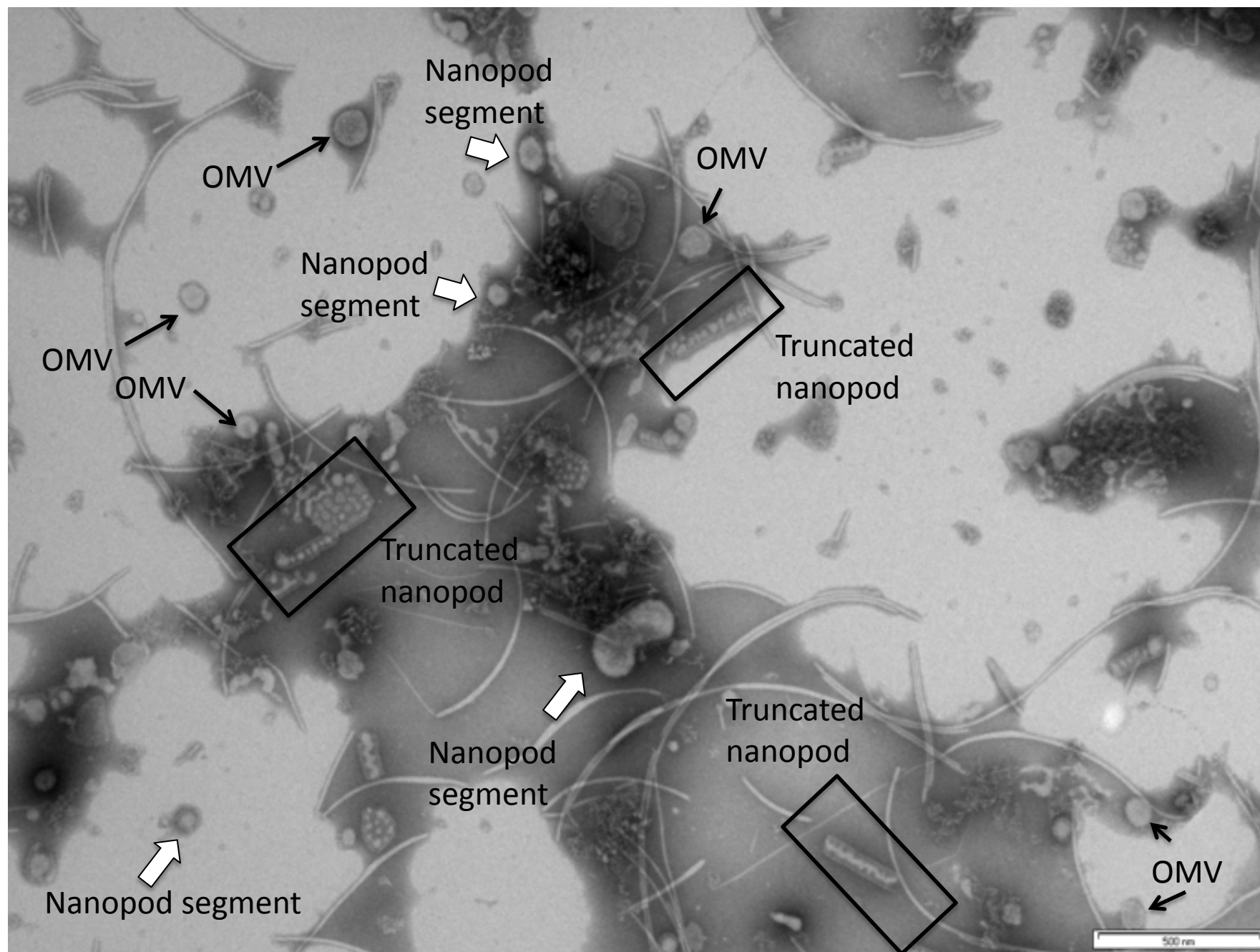

Figure S1\_H

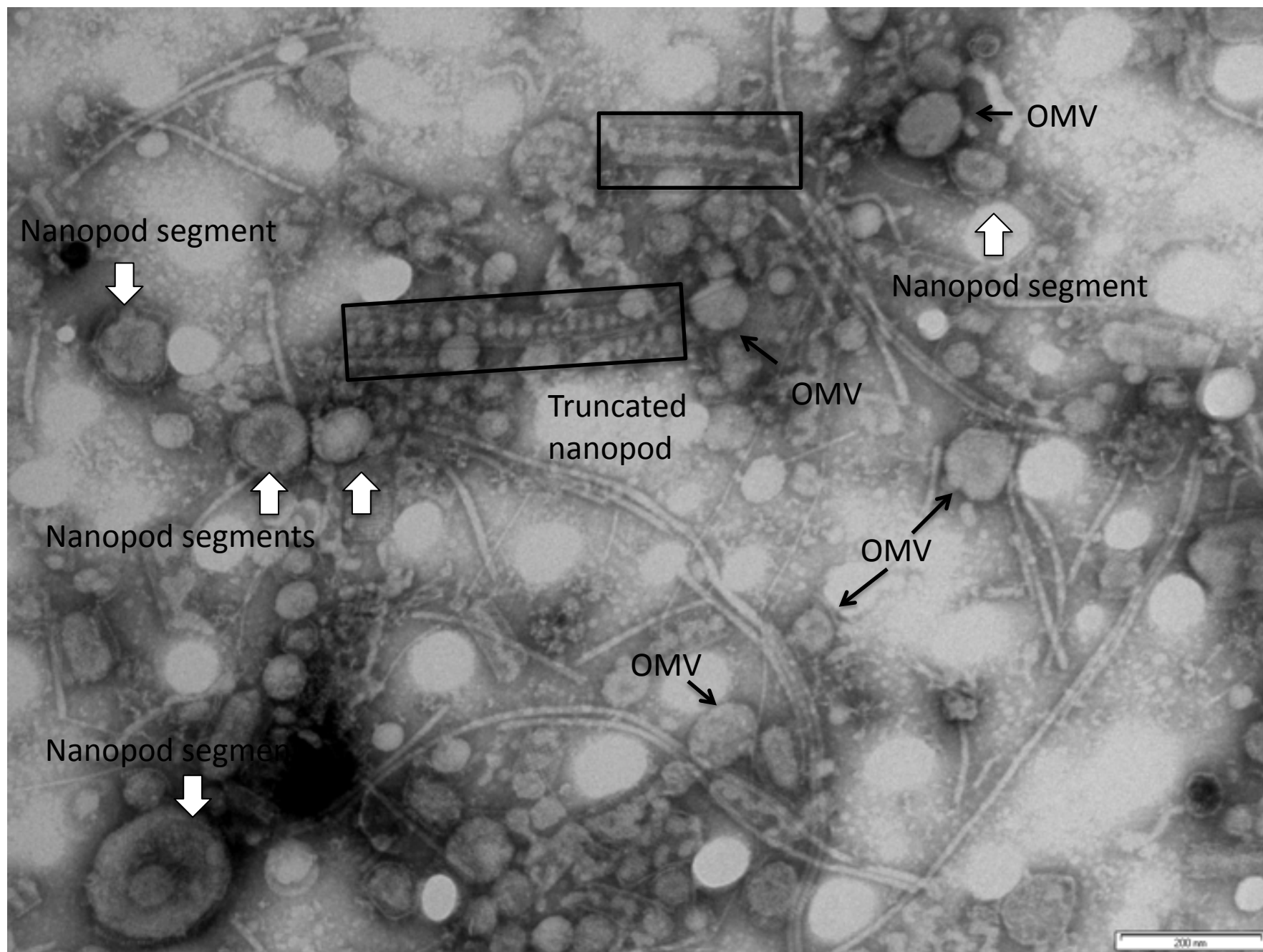

Figure S1\_I

Supplement: File S1 — Figure S1. Negatively stained cultures imaged by TEM showing extracellular material in phenanthrene-grown cultures of D. acidovorans Cs1-4 WT (Panels A-C), mutant M3 (Panels D-F ) and mutant M6 (Panels G-I). Panel A: Nanopods in the WT culture varying in length from ca. 50 nm (box) to ≥1500 nm (arrow). Panel B: Magnified view of WT nanopods showing linear structures (arrow) and segments (box). Panel C: Magnified view of WT nanopods showing cross-hatched surface structure indicative of paracrystalline S-layer. Panel D: Extracellular environment of mutant M3 containing OMV (boxes) and flagella (arrows) but devoid of nanopods. Panels E and F: Magnified view of OMV and flagella in the mutant M3 culture, with OMV ranging in size from ca. 20 nm to 100 nm. Panels G and H: Extracellular environment of mutant M6 containing nanopods (boxes), OMV (circles) and flagella (arrows). Nanopods were sparse compared to the WT, and truncated with none more than ca. 500 nm in length. Panel I: Magnified view of mutant M6 culture with truncated nanopods (boxes), OMV (circles) and flagella (arrows). (PDF) [file pone.0092143.s001.pdf]
