# Supplementary material for: Effects of Outer Membrane Vesicle Formation, Surface-Layer Production and Nanopod Development on the Metabolism of Phenanthrene by Delftia acidovorans Cs1-4
Source: PLoS One. 2014 Mar 18;9(3):e92143. doi: 10.1371/journal.pone.0092143 (PMC3958437; doi:10.1371/journal.pone.0092143)
Supplement: Table S1 — Delftia acidovorans Cs1-4 strains used in this study. (DOCX) [file pone.0092143.s002.docx]

Table S1. *Delftia* *acidovorans* Cs1-4 strains used in this study

| **Strain** | **Relevant characteristics** | | | **Source^a^** | | |
| --- | --- | --- | --- | --- | --- | --- |
| Wild type | Growth on phenanthrene, nanopod production | | Vacca et al. 2005 | |  |  |
| Flagella-free | Loss of flagella production by random transposon mutagenesis. Wild type with respect to growth on phenanthrene and nanopod production | | Shetty et al. 2011 | |  |  |
| Mutant M3 | Targeted insertional mutation of *npdA*, loss of surface layer protein (NpdA), retains OMV production, impaired growth on phenanthrene | | Shetty et al. 2011 | | |  |
| Mutant M6 | Loss of nanopod production and OMV production by random transposon mutagenesis, impaired growth on phenanthrene | Chen and Hickey, 2011 | | | | |

^a^ Vacca et al. 2005 Appl Environ Microbiol. 71:3797–3805; Shetty et al. 2011, PLoS One 6(6):e20725; Chen and Hickey 2011, [Front Microbiol.](http://www.ncbi.nlm.nih.gov/pubmed/22016746) 2:187.
